# Performance of polarization-sensitive neurons of the locust central complex at different degrees of polarization

**DOI:** 10.1007/s00359-022-01545-2

**Published:** 2022-02-14

**Authors:** Ronja Hensgen, Frederick Zittrell, Keram Pfeiffer, Uwe Homberg

**Affiliations:** 1 Department of Biology, Animal Physiology and Center for Mind Brain and Behavior (CMBB), Philipps-University of Marburg and Justus Liebig University of Giessen, 35032 Marburg, Germany; 2 Behavioral Physiology and Sociobiology (Zoology II), Biocenter, University of Würzburg, 97074 Würzburg, Germany

**Keywords:** Polarization vision, Central complex, Sky compass coding, Intracellular recordings, Desert locust

## Abstract

The polarization pattern of the sky is exploited by many insects for spatial orientation and navigation. It derives from Rayleigh scattering in the atmosphere and depends directly on the position of the sun. In the insect brain, the central complex (CX) houses neurons tuned to the angle of polarization (AoP), that together constitute an internal compass for celestial navigation. Polarized light is not only characterized by the AoP, but also by the degree of polarization (DoP), which can be highly variable, depending on sky conditions. Under a clear sky, the DoP of polarized sky light may reach up to 0.75 but is usually much lower especially when light is scattered by clouds or haze. To investigate how the polarization-processing network of the CX copes with low DoPs, we recorded intracellularly from neurons of the locust CX at different stages of processing, while stimulating with light of different DoPs. Significant responses to polarized light occurred down to DoPs of 0.05 indicating reliable coding of the AoP even at unfavorable sky conditions. Moreover, we found that the activity of neurons at the CX input stage may be strongly influenced by nearly unpolarized light, while the activity of downstream neurons appears less affected.

## Introduction

**Fig. 1:**
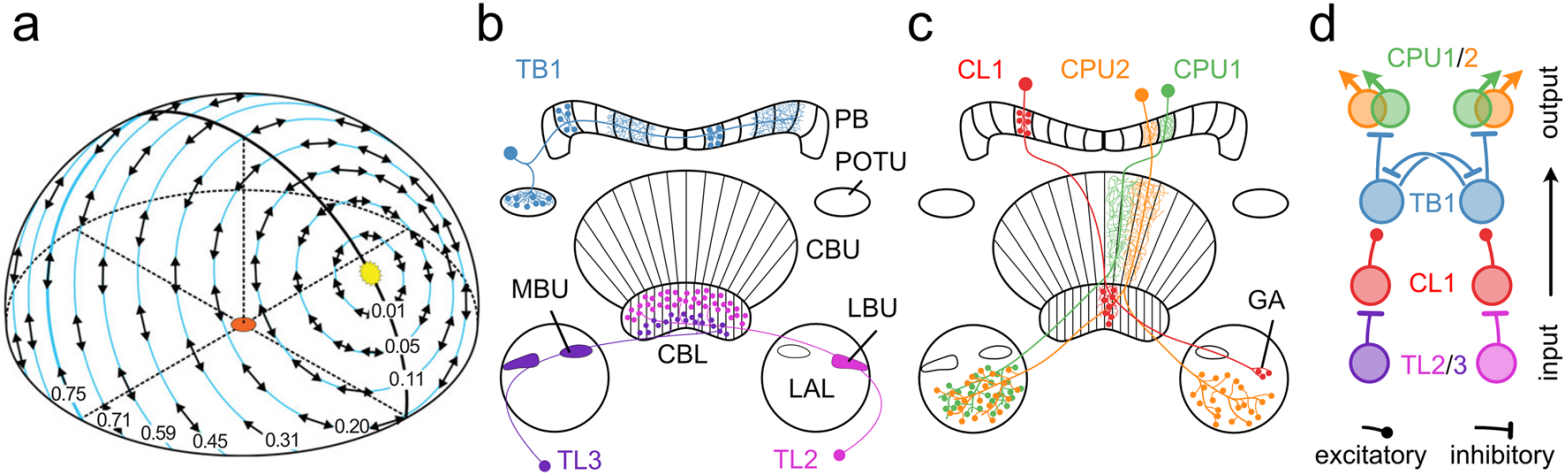
**a** Schematic representation of the polarization pattern of the sky as seen from the center of the sphere (orange) at a solar elevation of 40°. Double arrows indicate angles of polarization (AoP) that are arranged tangentially along concentric circles around the sun (yellow). Numbers indicate the degree of polarization (DoP). Under optimal atmospheric conditions the DoP increases with angular distance from the sun up to a maximum value of 0.75 at 90° from the sun. **b**, **c** Schematic illustration of tangential neurons (**b**) and columnar neurons (**c**) of the locust central complex. Fine branches indicate dendritic arborizations and small dots indicate axonal terminals. **b** TL2- and TL3 neurons provide input from the lateral bulb (LBU) and medial bulb (MBU) to the lower division of the central body (CBL). TB1 neurons connect the posterior optic tubercle (POTU) to the protocerebral bridge (PB). **c** CL1a neurons connect the CBL to the PB and the gall (GA). CPU1- and CPU2 neurons connect the PB to the upper division of the central body (CBU) and the lateral accessory lobes (LAL). **d** Putative processing hierarchy of the major types of polarization-sensitive neurons shown in **b**, **c**. TL2/TL3 neurons provide input to the central complex by synapsing onto intermediate-stage CL1a neurons. CL1a neurons transmit the information to TB1 neurons. Here, opponently tuned neurons inhibit each other and finally synapse onto CPU1/CPU2 output neurons. **a** From Homberg et al. (2011) and Pfeiffer et al. (2011), **b**, **c** modified from Pegel et al. (2019) and Zittrell et al. (2020). **d** modified from Bockhorst and Homberg (2017)

Spatial orientation and navigation require the perception and integration of environmental stimuli. For estimating spatial directions, many animals rely on sky compass cues, including celestial bodies such as the sun or moon, the chromatic gradient and the polarization pattern of the sky. Linear polarization of skylight mainly derives from Rayleigh scattering in the atmosphere (Strutt 1871) and results in a polarization pattern across the sky that directly depends on the position of the sun or moon (Fig. 1a). Orientation to polarized light has been demonstrated for several insect species in the field (honey bees, *Apis mellifera*: von Frisch 1949; Evangelista et al. 2014; desert ants, *Cataglyphis fortis*: Sommer and Wehner 2005; dung beetles, *Scarabaeus satyrus*: Dacke et al. 2013) and in the laboratory (desert locusts, *Schistocerca gregaria*: Mappes and Homberg 2004; field crickets, *Gryllus campestris*: Brunner and Labhart 1987; monarch butterflies, *Danaus plexippus*: Reppert et al. 2004). The neural pathways that mediate transmission of polarization information from the eye to the central brain have been studied particularly well in locusts (Homberg et al. 2003, 2011; Kinoshita et al. 2007), crickets (Labhart 1988; Sakura et al. 2007; Labhart et al. 2001) and fruit flies (Hardcastle et al. 2021) but also in other insects including monarch butterflies (Heinze and Reppert 2011) and dung beetles (el Jundi et al. 2015). Specialized photoreceptors of a small, dorsal region of the compound eye, the dorsal rim area (DRA), are particularly sensitive to the oscillation angle of polarized light (Labhart and Meyer 1999). Signals from dorsal rim photoreceptors are transmitted via the optic lobe, the anterior optic tubercle, and the bulb of the lateral complex to the central complex (CX) of the brain. The CX is an assembly of midline spanning neuropils, including the protocerebral bridge, the lower (CBL) and upper (CBU) division of the central body (corresponding to the ellipsoid body and the fan-shaped body in *Drosophila*, respectively), and the paired noduli. The CX houses a neural network signaling head-direction (Seelig and Jayaraman 2015; Green and Maimon 2018; Green et al. 2019; Pisokas et al. 2020; Hulse and Jayaraman 2020; Shiozaki et al. 2020) and as such integrates various sensory cues to generate appropriate behavioral output and guidance during navigation (Varga et al. 2017; Honkanen et al. 2021). The architecture of the CX is characterized by the projections of tangential and columnar neurons (Fig. 1b, c) that provide connectivity within the CX and between the CX and other brain regions (Hanesch et al. 1989; Heinze and Homberg 2008; Heinze et al. 2013; von Hadeln et al. 2020; Hulse et al. 2021).

As in other insects, many neurons of the CX of the locust *S. gregaria* are sensitive to the angle of polarization (AoP) of light from the sky. Physiological studies revealed a putative processing hierarchy for polarized light information in the CX (Fig. 1d; Heinze and Homberg 2009; Bockhorst and Homberg 2015): tangential neurons of the CBL (TL2, TL3 neurons, ER neurons or ring neurons in *Drosophila*) provide input from the bulbs of the lateral complex to the CX (Fig. 1b). Columnar neurons of the PB and CBL (CL1a neurons, E-PG neurons in *Drosophila*) transmit the signals to tangential neurons of the protocerebral bridge (TB1 neurons, ∆7 neurons in *Drosophila*) that signal onto columnar neurons of the PB and CBU (CPU neurons, PFL neurons in *Drosophila*). The latter provide output from the CX to the lateral accessory lobes (Fig. 1c). Consistent with the role of the CX as an internal compass, the orientation of the pattern of AoPs across the sky and the azimuth of a simulated sun are represented topographically in the neuronal activity across the protocerebral bridge (Heinze and Homberg 2007; Pegel et al. 2019; Zittrell et al. 2020).

Linearly polarized light is characterized, in addition to its AoP, by the degree of polarization (DoP) which indicates the percentage of polarized light within a light beam. The DoP depends on the angular distance from the sun and is lowest for direct sunlight (DoP = 0) and highest at 90° from the sun (DoP = 0.75 under optimal sky conditions; Fig. 1a). The DoP decreases under haze or clouds resulting in lower values. Several behavioral studies accounted for the natural occurrence of low DoPs by testing the performance of animals under matching conditions. For *A. mellifera* a detection threshold was proposed at a DoP between 0.07 and 0.1 (von Frisch 1967), and field crickets (*Gryllus campestris*) showed behavioral responses at DoPs even lower than 0.07 (Henze and Labhart 2007). Findings for the nocturnal dung beetle (*Scarabaeus satyrus*) indicated a behavioral threshold for polarized lunar skylight between 0.04 and 0.32 (Foster et al. 2019). These studies show that certain insect species can utilize polarization information for orientation even under highly unfavorable conditions. However, the majority of physiological experiments on neuronal responses to polarized light have been conducted with substantially higher DoPs (of 0.99). Only few studies examined the influence of low DoPs on neuronal responses. Among these are experiments performed in *G. campestris* that revealed a threshold DoP of 0.05 for polarization-opponent interneurons of the optic lobes (Labhart 1996) and an insensitivity of CX neurons of *G. bimaculatus* to changes in the DoP between 0.99 and 0.18 (Sakura et al. 2007). These findings fit the results from behavioral experiments. In contrast, neuronal responses of interneurons of the anterior optic tubercle of *S. gregaria* indicated a much higher DoP threshold of 0.3 and additionally demonstrated increasing neuronal inhibition upon stimulation with decreasing DoPs (Pfeiffer et al. 2011).

In this study, we investigated how different DoPs affect the responses of CX neurons of *S. gregaria* to polarized blue light*.* We show that reliable coding of AoPs is present in certain cell types down to DoPs of 0.05. Moreover, the activity of some neurons of the CX input is strongly affected by nearly unpolarized blue light, and this response is also mediated by the DRA.

## Materials and methods

### Animals and preparation

Male and female gregarious desert locusts were obtained from colonies reared at Philipps-Universität Marburg. Animals were kept at a constant temperature of 28 °C under a 12 h:12 h light/dark cycle. Animals were mounted onto a metal holder and legs and wings were cut off. A window was cut frontally into the head capsule, and fat tissue and air sacs were removed to get access to the brain. The esophagus was cut and the gut was removed via the abdomen, which was sealed afterwards with dental wax. The brain was stabilized from posterior with a small twisted metal wire inserted into the window of the head. Finally, the neural sheath of the brain was partly removed to allow penetration of the recording electrode. The brain was kept submerged in locust saline (Clements and May 1974) during preparation, recording and dissection.

### Electrophysiology

Intracellular recordings were performed with sharp microelectrodes drawn from borosilicate capillaries (Hilgenberg, Malsfeld, Germany), with a Flaming/Brown horizontal puller (P-97, Sutter Instrument, Novato, CA). The tip of the electrodes was loaded with 4% Neurobiotin (Vector Laboratories, Burlingame, CA) in 1 mol l^−1^ KCl and the shanks were loaded with 1 mol l^−1^ KCl. Signals were amplified 10 × with a BA-01 × amplifier (npi electronic instruments, Tamm, Germany), and monitored with a custom-built audio monitor (University of Marburg) and an oscilloscope (Hameg, Frankfurt/Main, Germany). Data were digitized with a Power1401-mkII (Cambridge Electronic Design, Cambridge, GB) and stored on a PC using Spike2 (Cambridge Electronic Design, Cambridge, UK) with a sampling rate of 20 kHz. After the recording, Neurobiotin was injected into the cell by application of a positive current of 0.5–2 nA for at least 2 min.

### Histology and image acquisition

Brains were dissected and fixed in 4% paraformaldehyde, 0.25% glutaraldehyde, and 2% saturated picric acid diluted in 0.1 mol l^−1^ phosphate buffered saline (PBS) at 4 °C overnight. Following rinses in PBS (4 × 15 min) brains were incubated in Cy3-conjugated streptavidin (1:1000) in PBS containing 0.3% Triton X-100 (PBT) at 4 °C for 3 days. Following rinses in PBT (2 × 20 min) and PBS (3 × 20 min) brains were dehydrated in an ascending ethanol series (30%, 50%, 70%, 90%, 95%, 100%, 15 min each) and cleared in a 1:1 mixture of 100% ethanol and methyl salicylate (Merck, Darmstadt, Germany) for 15 min and in pure methyl salicylate for 1 h. Finally, they were mounted in Permount (Fisher Scientific, Pittsburgh, PA) between two coverslips. Samples were scanned with a confocal laser scanning microscope (Leica, TCS SP5, Leica Microsystems, Wetzlar, Germany) with a 20 × immersion objective (HC PL APO 20 ×/0.75 Imm Corr CS2, Leica). A diode pumped solid state laser (561 nm) was used to excite Cy3. Scanning frequency was 400 Hz and the voxel size was 0.54 × 0.54 × 2.0 µm^3^.

### Stimulation

**Fig. 2:**
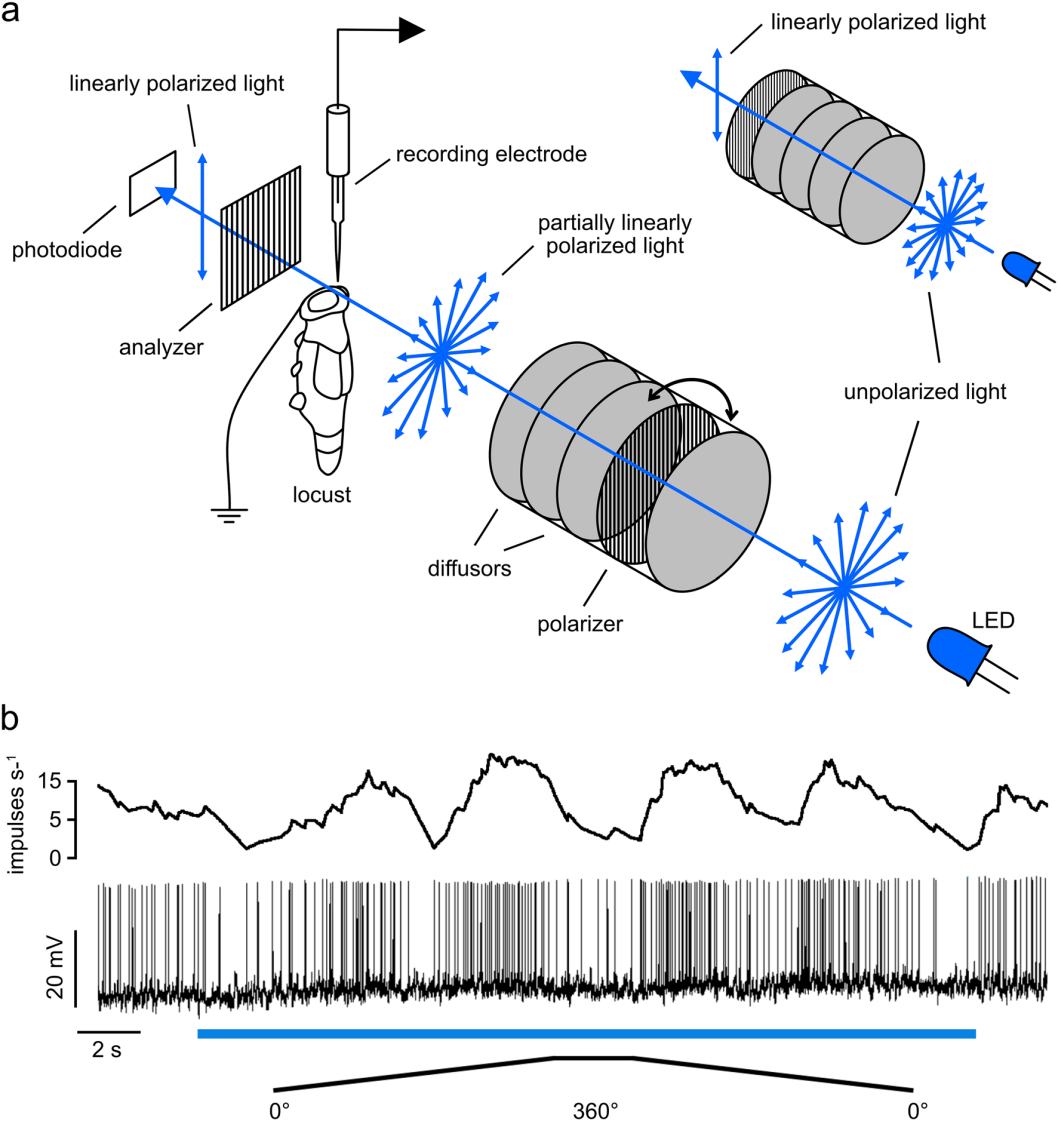
**a** Schematic illustration of the stimulus setup. Unpolarized light emitted by a light-emitting diode (LED) was linearly polarized by a polarizer. Diffusors were placed between the LED and the polarizer or between the polarizer and the animal to achieve different degrees of polarization (DoPs). With four diffusors between the LED and the polarizer (small image inset) maximally polarized light (DoP = 0.99) was generated. The degree and angle of polarization were measured via a photodiode/transimpedance amplifier placed behind a polarization filter. **b** Spike train showing the response of a CL1a neuron to two full rotations of the polarizer in clockwise and counterclockwise direction (0°–360°, 360°–0°). The blue bar indicates the time window during which polarized blue light was presented. The mean spiking frequency is indicated as moving average with a window size of 1 s above the spike train

Animals were stimulated from the zenith by light from a blue light emitting diode (LED; Oslon SSL 80, LDCQ7P, 452 nm, Osram Opto Semiconductors GmbH, Regensburg, Germany). The light was linearly polarized by a dichroic polarizer sheet (HNP’B, Polaroid, Cambridge, MA). To generate degrees of polarization between 0.002 and 0.9, diffusor sheets were placed in different combinations between the LED and the polarizer or between the animal and the polarizer (Fig. 2a). At the highest degree of polarization, all four diffusor sheets were placed between the LED and the polarizer, while at the lowest degree of polarization, the four diffusor sheets were placed between the animal and the polarizer. With the different arrangements, five stimuli could be tested: DoP = 0.99 (maximally polarized light, 1.9 × 10^13^ photons cm^−2^ s^−1^), DoP = 0.35 (1.6 × 10^13^ photons cm^−2^ s^−1^), DoP = 0.1 and DoP = 0.05 (1.5 × 10^13^ photons cm^−2^ s^−1^), and DoP = 0.002 (1.7 × 10^13^ photons cm^−2^ s^−1^). The stimuli covered a visual angle of 12.6°. To monitor the angle and the degree of polarization, a HNP’B polarization filter was placed in front of an OPT101 photodiode/transimpedance amplifier (Texas Instruments, Dallas, TX) positioned close to the animal’s head (Fig. 2a). The output of the OPT101 was continuously recorded via the digitizer. The stimulation device rotated 360° clockwise and counterclockwise at 40°/s. In four recordings from TL neurons, we painted the DRAs of the animal during the recording with acrylic black paint using a fine paintbrush. Removing the paint was also done while recording and was either done with forceps or with a paintbrush, depending on whether the paint was already dry or not.

### Data evaluation

Recording data were only analyzed when the recorded neuron was successfully labeled and the stained cell type unequivocally identified. Physiological data were preprocessed using Spike2 and exported as mat-files for further analysis in MATLAB (Version 2020a, The MathWorks, Natick, MA, USA) using custom scripts. Circular histograms were created with the CircHist package (Zittrell 2019). Confocal image stacks were processed in Amira 5.6 (ThermoFisher Scientific, Waltham, MA; RRID:SCR_007353). Images showing raw data were exported from Spike2 and processed with Affinity photo and Affinity Designer (Serif, Nottingham, UK; RRID:SCR_016951). Diagrams were generated with Microsoft Excel or MATLAB and were exported to Affinity photo to create figure panels.

#### Background activity

Owing to fluctuations of background activity (BA) in some neurons we calculated the BA for comparison with firing activity during stimulation within a time window of 5 s preceding the respective stimulus. Spikes were binned in 1 s bins and these spike counts were used to calculate the median and the 2.5th and 97.5th percentile of BA.

#### Stimulus responsiveness

We used linear-circular correlation analysis (*CircStat*; Berens 2009) to determine whether the modulation of spike rate was correlated to changes in AoP. Time points of action potentials during each 360° rotation were assigned to the respective orientation of the polarizer and these angles were doubled to allow using circular statistics on these axial data (Zar 1999). The resulting angles were averaged and the result was halved, yielding the preferred AoP (*Ф*_max_) in circular space. At least one clockwise and one counterclockwise rotation of the polarizer were included to measure the responsiveness to a stimulus with a particular DoP. To determine a correlation between firing rate and AoP, spike angles were binned in 10° bins and counts were tested for correlation with bin angles. A resulting *P* value < 0.05 indicated significant modulation by AoP. To specify properties of significant responses, we calculated the mean resultant vector length* r* and the response amplitude *A*. The vector length *r* describes the directedness of the response and ranges from 0 to unity, with unity indicating that all vectors are of the same direction (Batschelet 1981). It was calculated with the *CircStat* toolbox (Berens 2009). *A* describes the absolute amplitude of spike frequency modulation during stimulation, with higher *A* values indicating stronger modulation. *A* was calculated as follows according to Labhart (1996) and Pfeiffer et al. (2011):

$$A= \sum_{i=1}^{i=18}\left| {n}_{i}-\overline{n }\right|,$$

where *n*_*i*_ is the number of spikes in bin *i* and $\overline{n }$ is the number of spikes during the 360° rotation divided by the number of bins. Firing rates at *Ф*_max_ and *Ф*_min_ were estimated by fitting a bimodal von Mises distribution model to the binned data (Fitak and Johnsen 2017) and taking the model’s firing rate at the respective angles.

#### Regression analysis

To test whether modulation amplitude *A*, length of the mean vector *r*, and mean spiking activity were dependent on the DoP, respective data were pooled and tested for linear regression. Only cells that were stimulated with at least three different DoPs were included for this analysis. If the resulting residuals were not normally distributed (based on the Lilliefors test), the data were logarithmically transformed and the regression was done again. If the residuals of this regression were not normally distributed, the two linear models were compared regarding their *R*^*2*^ values and the one with the higher value was chosen.

#### Threshold for reliable coding of the AoP

To estimate the DoP threshold for reliable coding of the AoP we compared the mean resultant vector length *r* obtained during the stimulus with the upper 95% confidence limit of *r* obtained without stimulation (Pfeiffer et al. 2011). We defined the threshold as the lowest DoP at which the *r* values of all responses exceeded the upper 95% confidence limit of the estimated average *r* value of the no-stimulus controls.

## Results

We recorded intracellularly from 49 AoP-sensitive neurons in the CX, including 8 TL2 neurons, 5 TL3 neurons, 14 CL1a neurons, 10 TB1 neurons, 8 CPU1 neurons, and 4 CPU2 neurons. We investigated the influence of blue light with different DoPs on the mean vector length *r,* the response amplitude *A,* and the firing activity of the neurons. Based on the data, we determined a DoP threshold for reliable AoP signaling.

The recorded neurons showed characteristic sinusoidal modulation of activity during 360° rotation of the polarizer (Fig. 2b). The AoP that results in maximal activity during stimulus presentation is referred to as preferred AoP (or preferred *E*-vector orientation, *Ф*_max_). The AoP perpendicular to the *Ф*_max_ is called anti-preferred AoP (*Ф*_min_).

### Threshold for reliably signaling the angle of polarization

**Fig. 3:**
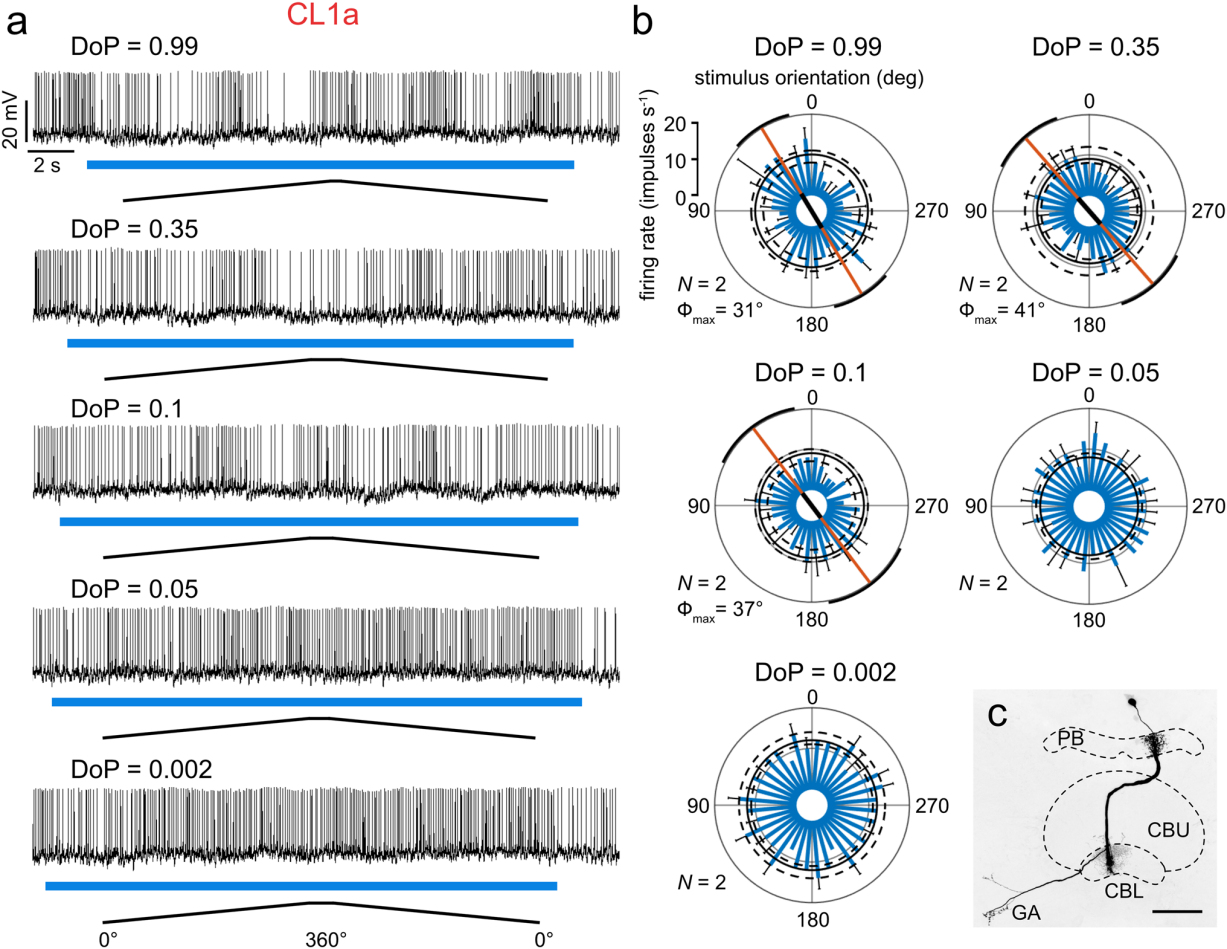
**a** Spike trains of a CL1a neuron in response to full clockwise- and counterclockwise polarizer rotations when stimulated with different degrees of polarization (DoP). The duration of the light stimulus is indicated by the blue bars. Ramps indicate 360° rotations of the polarizer, the angle of polarization (AoP) is not indicated by the ramps, as it is shifted depending on the arrangement of diffusors. **b** Circular histograms showing the firing rate (blue bars) during two polarizer rotations at five different DoPs. Black bars indicate standard deviations. If the firing rate was statistically significantly correlated with the AoP, the orange line indicates the preferred angle of polarization with the black portion indicating the resultant vector length. Black circles indicate median background activity (solid line) and the lower and upper 2.5 percentile (dashed lines) of the background activity. **c** Projection view of the recorded CL1a neuron with arborizations in the protocerebral bridge (PB), the lower division (CBL) of the central body and the gall (GA). CBU, upper division of the central body. Scale bar = 50 µm

All recordings (*N* = 49) included stimulation with blue light of the highest DoP (0.99). Depending on recording stability and duration additional stimuli were tested (DoP = 0.35, *N* = 41; DoP = 0.1, *N* = 34; DoP = 0.05, *N* = 33; DoP = 0.002, *N* = 45). Figure 3 illustrates the responses of a CL1a neuron that was tested with all five stimuli.

**Fig. 4:**
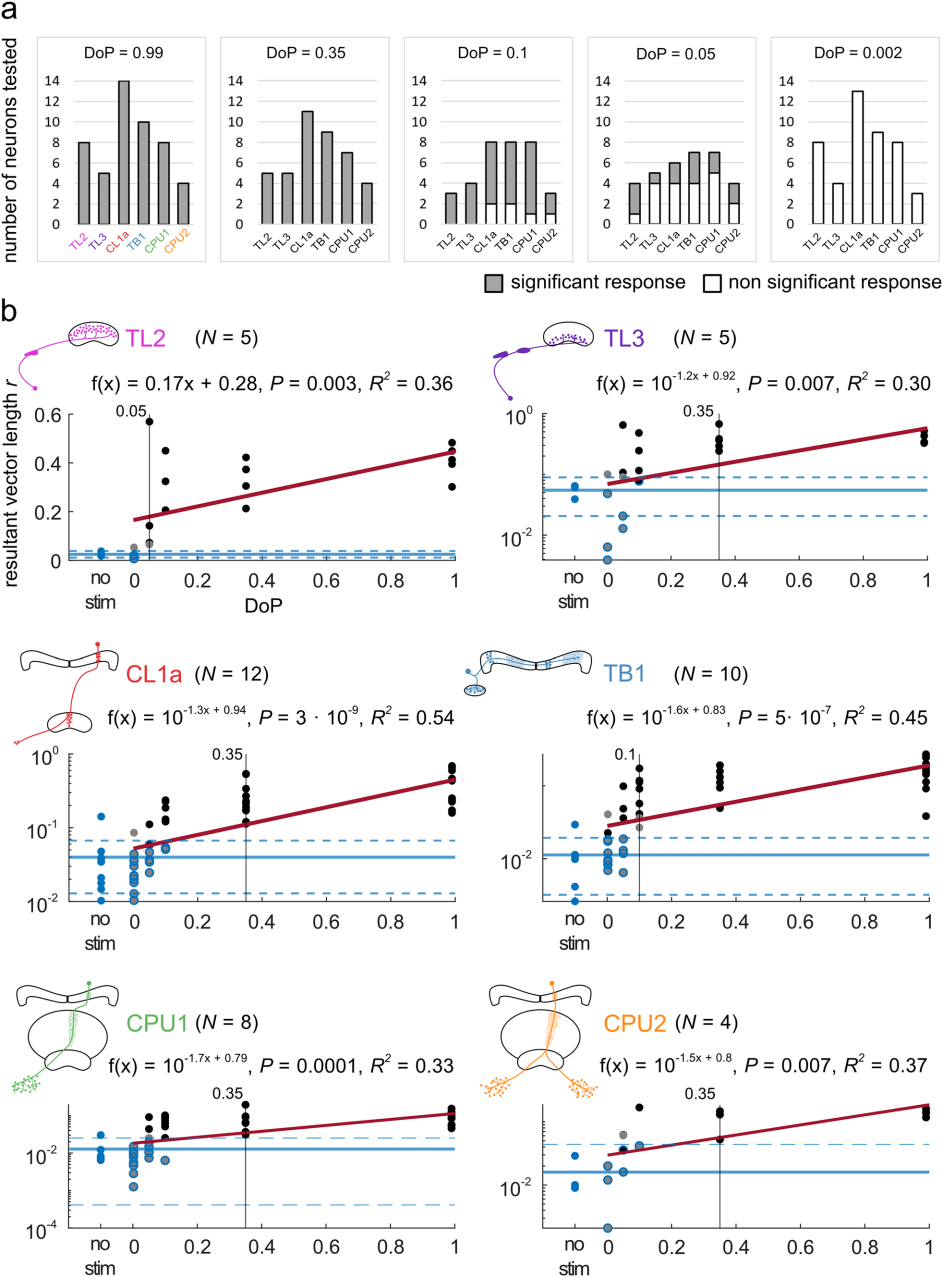
**a** Summary of significant (grey portion of bars) versus non-significant (white portion of bars) responses of different cell types at different degrees of polarization (DoP). All neurons responded significantly at DoPs of 0.99 and 0.35. At DoPs of 0.1 and 0.05 some neurons of each cell type showed significant responses, whereas others did not respond. At a DoP of 0.002 no neuron showed a significant response. **b** The mean vector length *r*, i.e., the directedness of responses increased with increasing DoP in all cell types. The regression lines are shown in red. The solid blue lines indicate the means of the no-stimulus data, the dashed blue lines indicate the respective lower and the upper 95% confidence limits. Vertical black lines mark the stimulus at which the *r* values of all responses exceed the upper 95% confidence limit of the estimated average *r* value of the no-stimulus controls. Blue dots are no-stimulus data points, grey dots are non-significant responses, grey dots with blue outline indicate non-significant responses that lie within the confident limits of the no-stimulus data. Black dots are significant-responses. Black dots with blue outline indicate significant-responses that lie within the confident limits of the no-stimulus data

All neurons (TL2 = 5, TL3 = 5, CL1a = 11, TB1 = 9, CPU = 11) tested with polarized blue light at a DoP of 0.35 showed a significant modulation of firing activity by AoP (Fig. 4a). All three TL2 neurons and all four TL3 neurons tested with a DoP of 0.1 still responded significantly to the stimulus. Most of the CL1a neurons (6 out of 8), TB1 neurons (6 out of 8), CPU1 neurons (7 out of 8), and CPU2 neurons (2 out of 3) also showed significant responses at 0.1 DoP. At a DoP of 0.05 less than half of the tested neurons responded significantly to the stimulus (TL2, 3 out of 4; TL3, 1 out of 5; CL1a, 2 out of 6; TB1, 3 out of 7; CPU1, 2 out of 7; CPU2, 2 out of 4). The lowest DoP of 0.002, which should equal unpolarized light, did not elicit a significant response in any of the neurons tested. These results (summarized in Fig. 4a) point to a threshold for reliable coding of the AoP between a DoP of 0.1 and 0.05.

Pfeiffer et al. (2011) used the mean vector length *r* to calculate the threshold for reliable AoP coding (see “Materials and Methods”). In TL2 neurons all *r* values at DoP values ≥ 0.05 exceeded the upper 95% confidence level (Fig. 4b). TB1 neurons showed reliable coding at DoP values ≥ 0.1. In all other cell types stimuli with a DoP ≥ 0.35 resulted in reliable coding of the AoP (Fig. 4b).

### Response amplitude and firing activity at different degrees of polarization

**Fig. 5:**
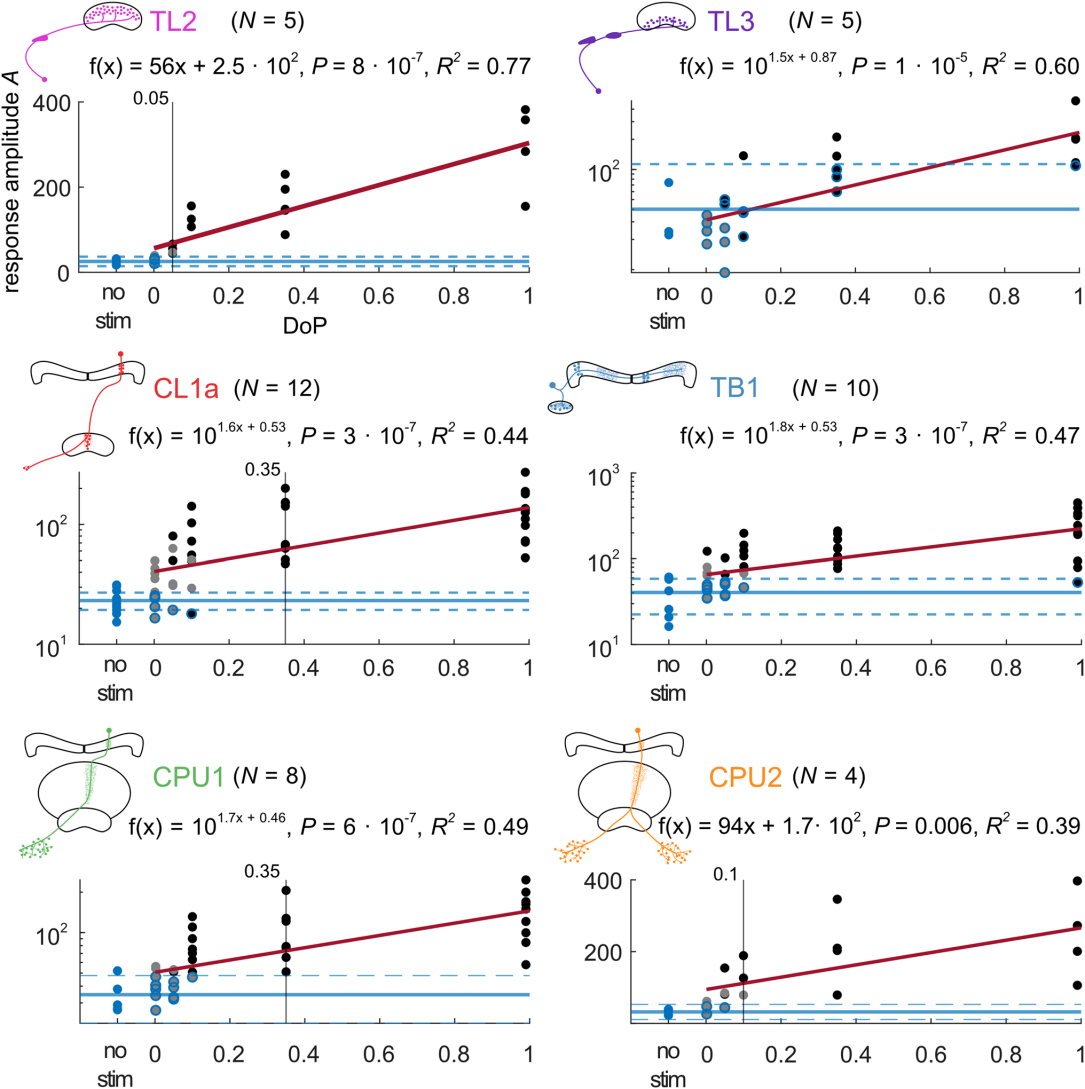
The absolute response amplitude *A* increases with increasing degree of polarization in all cell types. The regression lines are shown in red. The solid blue lines indicate the means of the no-stimulus data, the dashed blue lines indicate the respective lower and the upper 95% confidence limits. Blue dots are no-stimulus data points, grey dots are non-significant responses, grey dots with blue outline indicate non-significant responses that lie within the confident limits of the no-stimulus data. Black dots are significant responses. Black dots with blue outline indicate significant responses that lie within the confident limits of the no-stimulus data. Vertical black lines mark the stimulus at which the *A* values of all responses exceed the upper 95% confidence limit of the estimated average *A* value of the no-stimulus controls

**Fig. 6:**
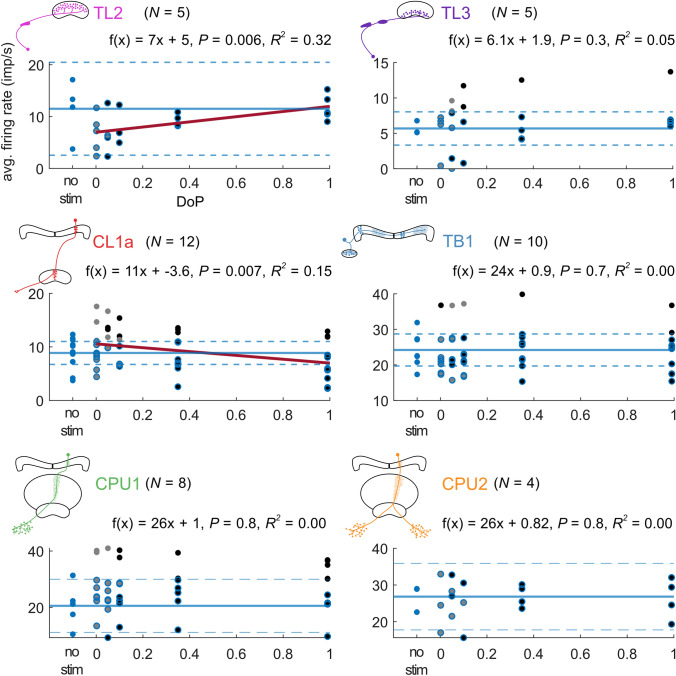
The average firing activity during stimulation with a rotating polarizer was positively correlated with the degree of polarization (DoP) in TL2 neurons and negatively correlated with the DoP in CL1a neurons. In TL3, TB1 and CPU neurons the average firing activity was not correlated with the DoP. The regression lines are shown in red. The solid blue lines indicate the mean of the no-stimulus data, the dashed blue lines indicate the respective lower and the upper 95% confidence limits. Blue dots are no-stimulus data points, grey dots are non-significant responses, grey dots with blue outline indicate non-significant responses that lie below the upper confidence limit of the no-stimulus data. Black dots are significant-responses. Black dots with blue outline indicate significant-responses that lie below the upper confident limit of the no-stimulus data

**Fig. 7:**
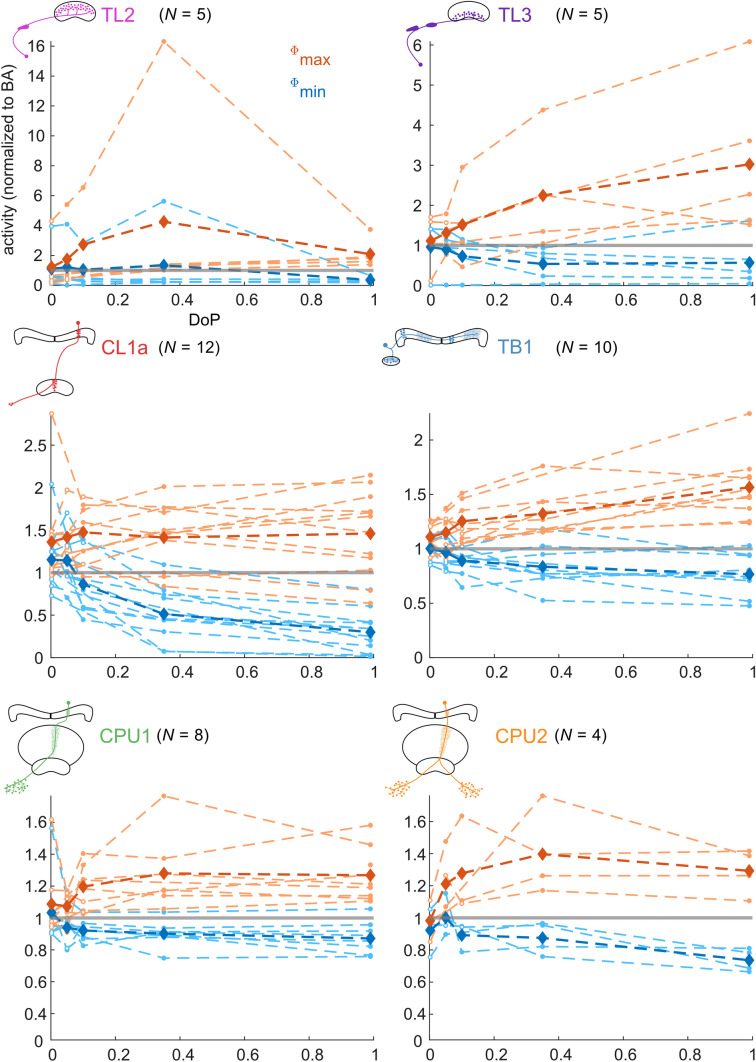
Activity of individual neurons at *Ф*_max_ (orange) and *Ф*_min_ (blue) during responses to a rotating polarizer at different degrees of polarization (DoP). Activity is normalized to background activity of a 5 s interval (median value of 1-s-binned spike rate averages) preceding each stimulus. The grey lines indicate background activity. Dots indicate significant responses, whereas circles indicate non-significant responses. The bold lines indicate the averaged activity of all neurons at *Ф*_max_ and *Ф*_min_, respectively

**Fig. 8:**
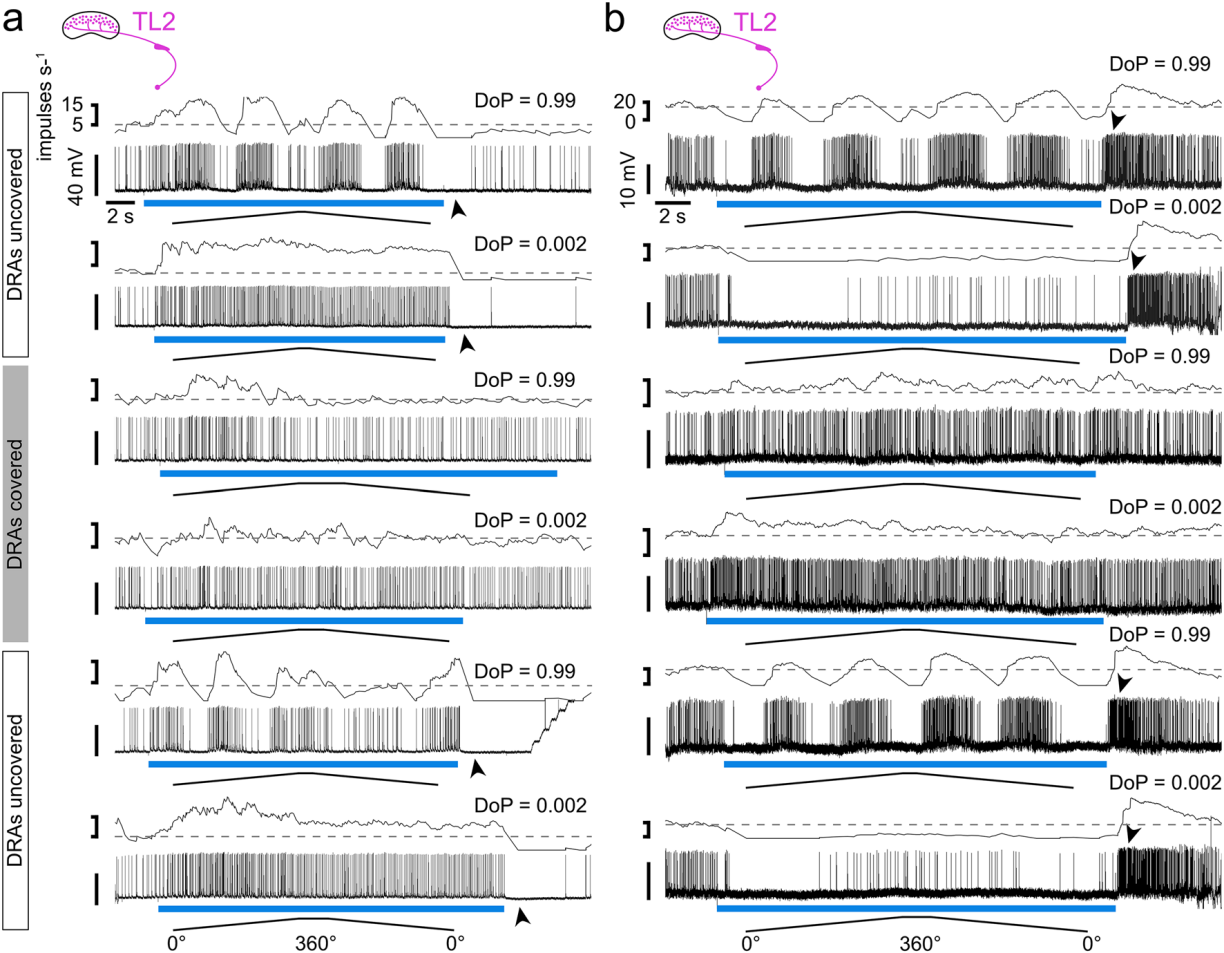
Responses of two different TL2 neurons to polarizer rotations with the lowest degree of polarization (DoP = 0.002) and the highest degree of polarization (DoP = 0.99). The blue bars indicate the time windows during which polarized blue light was presented. Ramps indicate 360° rotations of the polarizer. Dashed lines indicate median background activity during 5 s preceding each stimulus. While the neuron in **a** is excited by low DoPs, the neuron in **b** gets inhibited. Covering the DRA of both eyes abolished the polarization response, the excitation (**a**) or inhibition (**b**) during low DoPs, and the inhibitory rebound (**a**) or excitatory rebound (**b**) at lights off (arrowheads). Uncovering the DRAs restored the responses

The response amplitude *A* was positively correlated with increasing DoPs in all types of neuron tested (Fig. 5). Testing for linear regression revealed that the dependence of *A* on the DoP in TL2 neurons and CPU2 neurons was best described when using non-logarithmically transformed data, whereas the dependence of *A* on the DoP in the other neurons was best described by a linear model based on logarithmically transformed data (see “Materials and Methods”, Fig. 5). This indicates that the relationship between the response amplitude *A* and the DoP is linear in TL2 neurons and CPU2 neurons but logarithmic in the remaining cell types. However, individual neurons of each type could show an either linear or logarithmic relationship between the response amplitude *A* and the DoP (not shown). Similar to Pfeiffer et al. (2011) we calculated whether the mean spiking activity during a 360° rotation of the polarizer was influenced by the presented DoP. We found that the mean spiking activity was positively correlated with increasing DoPs in TL2 neurons, negatively correlated in CL1a neurons and not linearly correlated in TL3-, TB1- and CPU neurons (Fig. 6). To further explore the cell-type-specific results, we calculated the minimum and maximum activity of neurons at *Ф*_max_ and *Ф*_min_ for different DoPs (Fig. 7). The results show that both, inhibition and excitation, increased in all types of neuron with an increase in DoP, and that activity during low DoPs was clustered around background activity in TL3, TB1 and CPU neurons (Fig. 7). In contrast, activity of CL1a neurons at *Ф*_max_ and *Ф*_min_ at low DoPs was increased above background activity, whereas in TL2 neurons activity at low DoPs was lower than background activity, except for one cell (Fig. 7, TL2, Fig. 8a).

### Influence of unpolarized blue light on firing activity

The activity of six neurons (5 TL2, 1 TL3) was strongly affected by unpolarized blue light. Reducing the DoP revealed an overall inhibition or excitation of the neurons during stimulus presentation with strongest effects at the lowest DoP of 0.002 (Fig. 8). Excitatory and inhibitory responses were followed by rebound inhibition or excitation, respectively, at stimulus offset. These effects were observed primarily in TL neurons, especially TL2 cells, but could also be observed, however to a lesser extent, in CL1a-, TB1- and CPU neurons.

Five out of eight TL2 neurons were inhibited and two were excited at 0.002 DoP, and among four TL3 neurons one was inhibited. The strength of inhibition and excitation varied between individual cells and could be very pronounced or rather mild. The remaining neurons (one TL2 and three TL3) did not show obvious changes in firing activity upon stimulation with unpolarized blue light.

The strong excitatory response of a TL2 neuron to stimulation with the lowest DoP as well as the responses to higher degrees of polarization were followed by strong inhibition upon stimulus offset which lasted up to 20 s (Fig. 8a, arrowheads). These inhibitions at lights off were abolished after the DRAs were covered with black paint (Fig. 8a) but were restored when uncovering the DRAs again (Fig. 8a). Painting the DRAs resulted, in addition, in higher overall activity of the neuron, perhaps owing to the lack of inhibition following each stimulus. The opposite response, again in a TL2 neuron, is illustrated in Fig. 8b. Here the TL2 neuron responded to low DoPs with inhibition and rebound excitation at lights off. Both responses were abolished when the DRAs were covered (Fig. 8b). Uncovering the DRAs restored the polarization response at high DoPs, the inhibition at low DoPs (unpolarized blue light) and the excitation at lights off (Fig. 8b). Covering the DRA with black paint during the recording from one TL3 neuron and another TL2 neuron (data not shown) showed the same results.

**Fig. 9:**
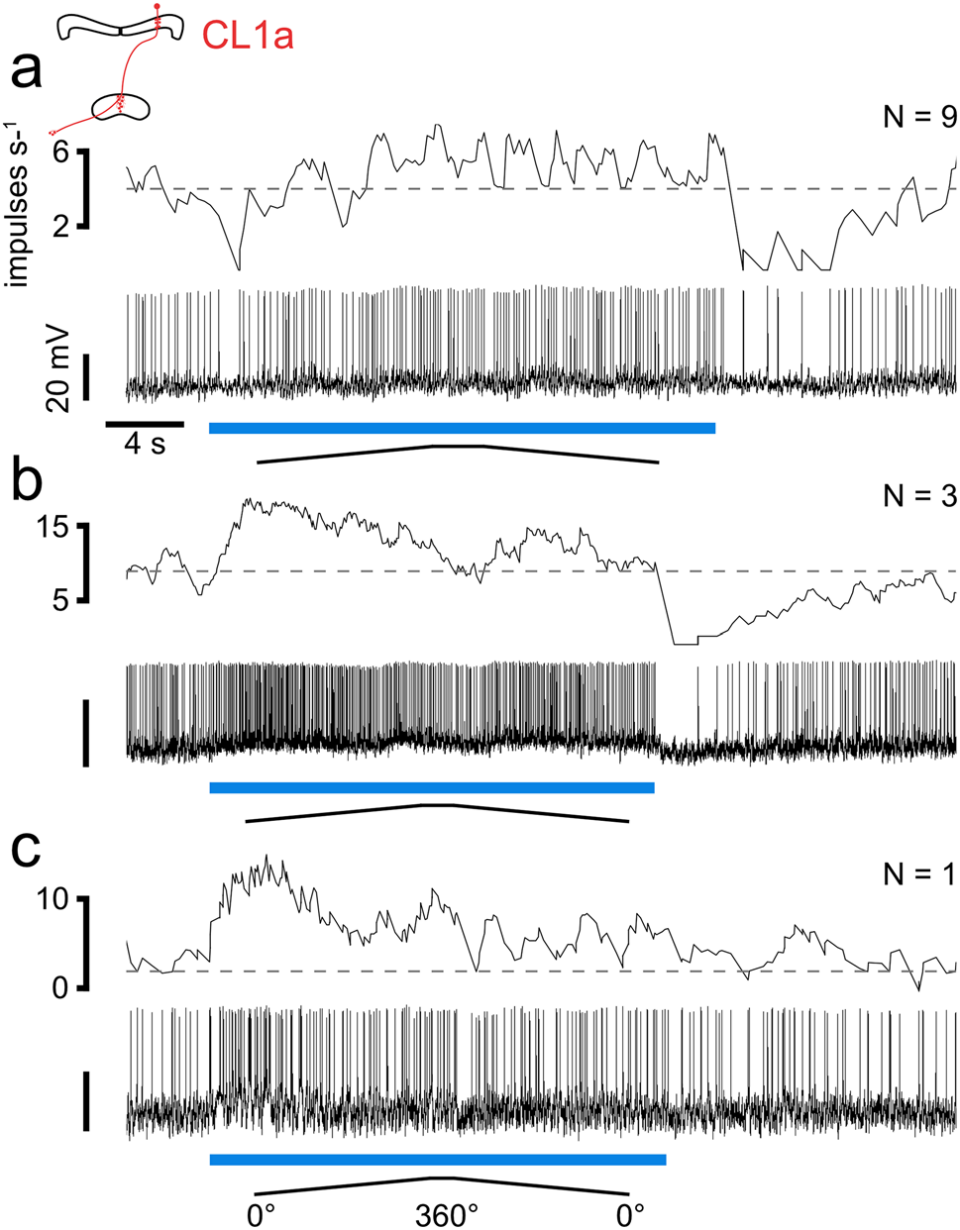
Responses of three different CL1a neurons to the lowest degree of polarization (DoP = 0.002). The blue bars indicate the time windows during which polarized blue light was presented. Ramps indicate 360° rotations of the polarizer. Dashed lines indicate median background activity during 5 s preceding each stimulus. **a** This CL1a neuron responded with slightly elevated activity preceded by phasic inhibition at lights on. Phasic rebound inhibition occurs at lights off. **b** This CL1a neuron responded with excitation to the stimulus, followed by rebound inhibition at lights off. **c** This CL1a neuron displayed phasic excitation at lights on. *N* indicates how many of the recorded cells showed similar responses to the lowest DoP

In 12 out of 13 CL1a neurons we observed phasic inhibition after stimulus offset (lights off) that varied in strength. Nine of these neurons displayed phasic inhibition also at stimulus onset (lights on) that gave way to slightly elevated activity (Fig. 9a). Three neurons did not show phasic lights on inhibition but only excitation during stimulation with the lowest DoP (Fig. 9b). Although the strength and duration of phasic inhibition at lights on, the following sustained excitation, and the rebound inhibition upon lights off varied between individual neurons, only one CL1a neuron showed a completely different response characterized by phasic excitation upon lights on (Fig. 9c).

**Fig. 10:**
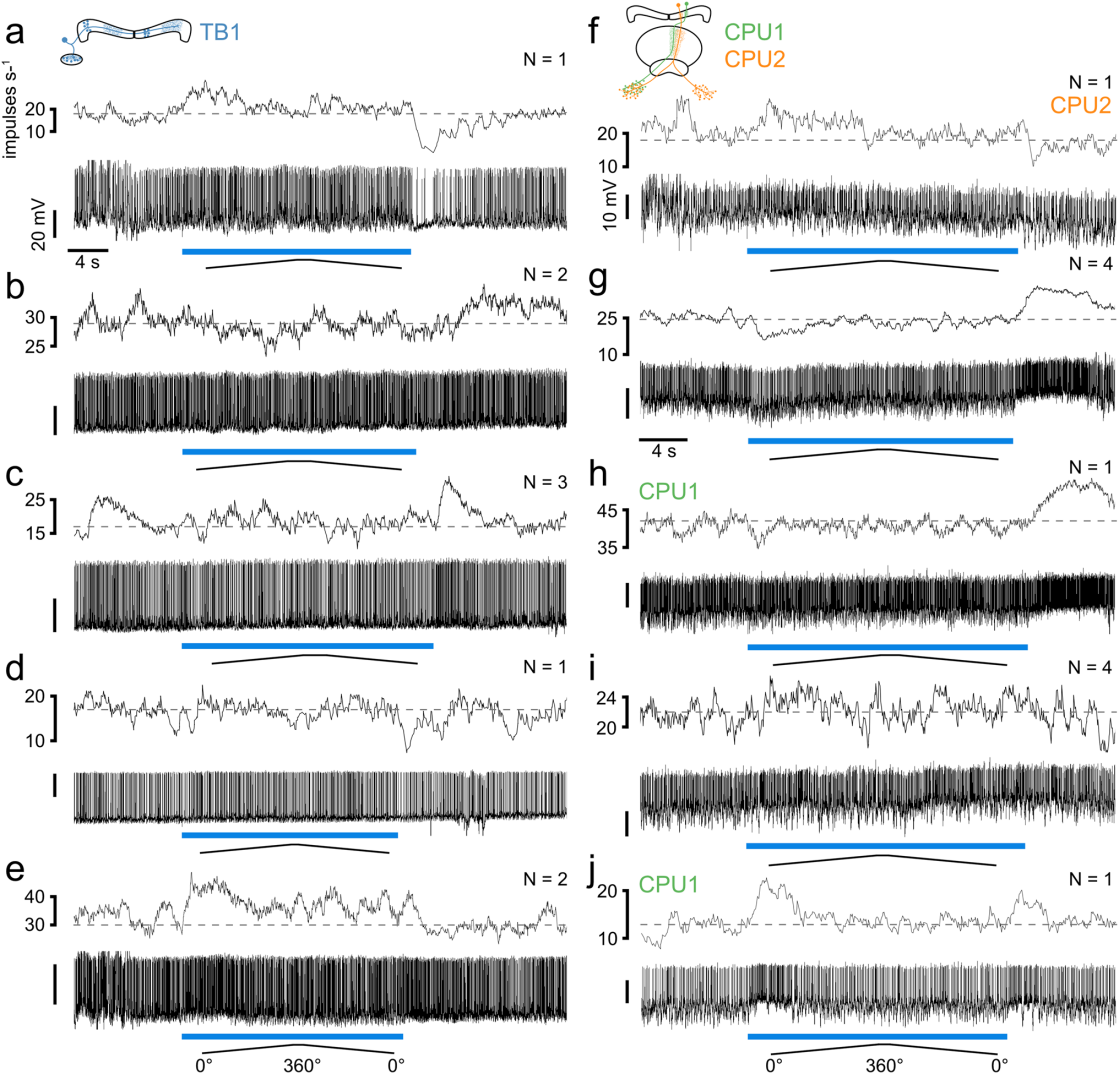
Responses of five different TB1 neurons (**a**–**e**) and five different CPU neurons (**f**–**j**) to the lowest degree of polarization (DoP = 0.002). The blue bars indicate the time windows during which polarized blue light was presented. Ramps indicate 360° rotations of the polarizer. Dashed lines indicate median background activity during 5 s preceding each stimulus. **a** This TB1 neuron showed slight excitation during stimulus presentation and rebound inhibition at lights off.** b** This TB1 neuron showed slight inhibition during stimulus presentation followed by rebound excitation. **c** This TB1 neuron displayed phasic excitation at lights off. **d** This TB1 neuron displayed weak rebound inhibition at lights off. **e** This TB1 neuron showed slight excitation during stimulus presentation and lacked rebound inhibition. **f** This CPU2 neuron displayed slight excitation during stimulus presentation and slight inhibition at lights off. **g** This CPU1 neuron was slightly inhibited during stimulus presentation and rebound excitation occurred at lights off. **h** This CPU1 neuron displayed phasic excitation at lights off. **i** This CPU1 neuron showed no change in activity during stimulus presentation. **j** This CPU1 neuron displayed phasic excitation at lights on and at lights off. *N* indicates how many of the recorded cells showed similar responses to the lowest DoP

Nine TB1 neurons showed more variable responses. One neuron showed slight excitation followed by rebound inhibition (Fig. 10a). Two neurons showed slight inhibition during stimulation followed by rebound excitation (Fig. 10b), three neurons showed only excitation upon stimulus offset (Fig. 10c), and one neuron showed slight inhibition after stimulus offset (Fig. 10d). Two neurons showed slight excitation during stimulation but lacked rebound inhibition (Fig. 10e).

Of the 11 CPU neurons one CPU2 neuron showed slight excitation during stimulus presentation followed by rebound inhibition (Fig. 10f). Four CPU neurons (two CPU1, two CPU2) showed inhibition during stimulation with a DoP = 0.002 and rebound excitation upon stimulus offset (Fig. 10g). One CPU1 neuron displayed rebound excitation at lights off but no activity change during stimulation (Fig. 10h). Four CPU neurons (three CPU1, one CPU2) showed no obvious change in activity during stimulation (Fig. 10i). The remaining CPU1 neuron showed phasic excitation upon stimulus onset and rebound excitation upon stimulus offset (Fig. 10j).

## Discussion

### Threshold for reliable AoP signaling

Intracellular recordings from AoP-sensitive neurons of the locust CX revealed that AoP signaling in these neurons is reliable down to DoPs of 0.35 in TL3-, CL1a-, and CPU neurons, 0.1 in TB1 neurons and 0.05 in TL2 neurons. Because our estimation of reliable coding does not account for DoPs between the discrete values that were tested (0.35, 0.1 and 0.05), and based on the significance of responses of individual neurons, we assume that at least for TL3-, CL1a- and CPU neurons the actual threshold for AoP coding might be lower than the estimated threshold and might lie between 0.35 and 0.1. The low thresholds found in TL2 and TB1 neurons are similar to thresholds that have been determined for polarotactic behavior in honeybees, crickets, and dung beetles (von Frisch 1967; Henze and Labhart 2007; Foster et al. 2019) and for neuronal responses of polarization-opponent interneurons in the optic lobes of crickets (Labhart 1996). Prior to this study, neurons of the CX have been tested with different DoPs only in crickets (CNL neurons, Sakura et al. 2007). Those neurons are homologous to TL2/TL3 neurons in locusts. They showed responses to polarized light with modulation amplitudes independent of the DoP, ranging from 0.99 to 0.18. Lower DoPs, however, were not tested. In contrast to the data in crickets, the modulation amplitude in all types of locust CX neurons increased with increasing DoP (Fig. 5). This should be advantageous for encoding sun positions through matched-filter coding of sky polarization patterns as shown by Zittrell et al. (2020). Locust CX neurons integrate polarization information not only from the zenith but across the entire sky and respond best to polarization patterns that match a particular position of the sun. Because each point in the sky is not only characterized by polarization angle, but also by DoP depending on distance from the sun (Fig. 1a), both parameters should ideally be considered and integrated in coding of sky polarization patterns.

For two cell types of the anterior optic tubercle of the locust (LoTu1- and TuTu1 neurons), Pfeiffer et al. (2011) determined a DoP threshold for reliable AoP signaling of 0.3. This threshold is similar to the threshold we determined for TL3-, CL1a-, and CPU neurons but higher than the threshold determined for TL2 and TB1 neurons. When comparing these different types of neuron one has to take into account that LoTu1- and TuTu1 neurons are not directly involved in the polarization vision pathway to the CX, but rather provide integration between the right and left tubercle in the locust brain.

### Effect of unpolarized blue light on polarization-sensitive neurons

In LoTu1 neurons dorsally presented polarized blue light increases spiking activity irrespective of the angle of polarization, whereas dorsally presented unpolarized light decreases overall spiking activity (Pfeiffer et al. 2011). Because unpolarized light consists of all possible angles of polarization these results appeared puzzling and led to a hypothetical model of the underlying mechanisms. The authors suggested that the temporal and spatial pattern of histamine release by polarization-sensitive photoreceptors leads to inhibition and rebound excitation of lamina neurons which becomes visible in LoTu1 neurons. In TL neurons we found similar, but also opposite responses, with pronounced inhibition upon presentation of unpolarized blue light (Fig. 8b) in five cells, but also pronounced excitation upon stimulation with unpolarized blue light (Fig. 8a) in one cell. Although these responses are reminiscent of those described for LoTu1 neurons, we propose that the underlying mechanism is different. Whereas DRA-mediated inhibition and excitation in LoTu1 neurons are suggested to be driven by only one input, we suggest that TL2 and TL3 neurons receive inhibitory and excitatory input. This assumption is supported by polarization opponency in TL2 and TL3 neurons (Pegel et al. 2018) which would result from inhibitory input at *Ф*_min_ and excitatory input at *Ф*_max_. Pronounced inhibition or excitation at low DoP values in TL neurons might, therefore, result from unbalanced inhibitory and excitatory inputs leading to an overall excitation or inhibition when reducing the DoP. In accordance with the hypothetical model provided by Pfeiffer et al. (2011), unpolarized light would equally excite all polarization-sensitive photoreceptors and would lead to either inhibition or excitation in TL neurons depending on whether the inhibitory input outweighs the excitatory input or vice versa. Balanced inhibitory and excitatory input would result in unaltered neuronal activity upon presentation of low DoPs. Pfeiffer et al. (2011) plotted the average firing rate of LoTu1- and TuTu1 neurons over the DoP to illustrate the increase in average firing rate with increasing DoPs. We found a similar trend in TL2 neurons but for TL3 neurons we found no correlation (Fig. 6). These findings indicate that TL2 and TL3 neurons serve slightly different purposes.

The suppression of activity in LoTu1 neurons might prevent signaling of ambiguous information deriving from low DoPs (Pfeiffer et al. 2011). Unbalanced input to TL neurons that results in either pronounced excitation or inhibition might serve to modulate the TL network activity according to available stimuli, i.e. silencing the polarization-processing pathway when respective stimuli are absent, clearing the way for other navigational relevant stimuli, such as wind (Okubo et al. 2020) or proprioceptive feedback. Pfeiffer et al. (2011) assumed that the inhibition by low DoP in LoTu1- and TuTu1 neurons is caused by the same set of polarization-sensitive photoreceptors that signal relevant AoP stimuli. Here, we demonstrate that photoreceptors of the DRA, indeed, mediate the inhibitory and excitatory responses to dorsally presented unpolarized blue light in TL neurons (Fig. 8), whereas other eye regions have only a marginal, if any, effect.

Unpolarized blue light had similar but less prominent effects in the downstream cell types of the CX. In CL1a neurons consistent increase in firing rate during stimulus presentation corresponded to rebound inhibition after stimulus offset. This response pattern is opposite to that found in most TL2 neurons (Fig. 8b). In accordance with that, the average firing activity of CL1a neurons was negatively correlated with an increase in the DoP. All of these properties support the assumption that CL1a neurons are inhibited by the GABAergic TL neurons. Because the activity of CL1a neurons is likely modulated by global inhibition from many TL neurons, their excitation during low DoP is less pronounced likely by convergent input from many TL neurons, some of which are excited as well (Fig. 8a).

The responses in TB1- and CPU neurons to unpolarized blue light were weaker and more variable than those of CL1a neurons. This may be a result of mutual inhibition of heterolateral TB1 neurons proposed by Bockhorst and Homberg (2017) illustrated in Fig. 1d. Slight inhibition observed in several CPU neurons might point to a net inhibitory input to CPU neurons from CL1a neurons. In both, TB1- and CPU neurons, the average firing activity appears to be independent of the DoP.

## Conclusions

The data show that the CX in desert locusts is capable of reliable AoP coding and thus sky-compass dependent head-direction signaling even under highly unfavorable sky conditions. As our stimulus device only covered a visual angle of 12.5°, even lower effective degrees of polarization in the sky may suffice to generate head-direction signals by integration of inputs across the full sky as shown by Zittrell et al. (2020). This might allow to still exploit skylight polarization at a sky fully overcast by thin clouds showing effective degrees of polarization just above 0.05 (Labhart 1996, 1999).

## Abbreviations

AoP: Angle of polarization
CBL: Lower division of the central body
CBU: Upper division of the central body
CL neurons: Columnar neurons of the protocerebral bridge and lower division of the central body
CPU neurons: Columnar neurons of the protocerebral bridge and upper division of the central body
CX: Central complex
DRA: Dorsal rim area
DoP: Degree of polarization
TB neurons: Tangential neurons of the protocerebral bridge
TL neurons: Tangential neurons of the lower division of the central body

## Declarations

### Availability of data and materials

All data that support the findings of this study are available from the corresponding author.

### Code availability

MATLAB analysis code is available upon request.
